# Zoo agent’s measure in applying the five freedoms principles for animal welfare

**DOI:** 10.14202/vetworld.2017.1026-1034

**Published:** 2017-09-03

**Authors:** Argyo Demartoto, Robertus Bellarminus Soemanto, Siti Zunariyah

**Affiliations:** Department of Sociology, Faculty of Social and Political Sciences, Universitas Sebelas Maret, Surakarta, Indonesia

**Keywords:** agent, animal welfare, structure, the five freedoms, zoo

## Abstract

**Background:**

Animal welfare should be prioritized not only for the animal’s life sustainability but also for supporting the sustainability of living organism’s life on the earth. However, Indonesian people have not understood it yet, thereby still treating animals arbitrarily and not appreciating either domesticated or wild animals.

**Aim:**

This research aimed to analyze the zoo agent’s action in applying the five freedoms principle for animal welfare in Taman Satwa Taru Jurug (thereafter called TSTJ) or Surakarta Zoo and Gembira Loka Zoo (GLZ) of Yogyakarta Indonesia using Giddens structuration theory.

**Materials and Methods:**

The informants in this comparative study with explorative were organizers, visitors, and stakeholders of zoos selected using purposive sampling technique. The informants consisted of 19 persons: 8 from TSTJ (Code T) and 10 from GLZ (Code G) and representatives from Natural Resource Conservation Center of Central Java (Code B). Data were collected through observation, in-depth interview, and Focus Group Discussion and Documentation. Data were analyzed using an interactive model of analysis consisting of three components: Data reduction, data display, and conclusion drawing. Data validation was carried out using method and data source triangulations.

**Results:**

Food, nutrition, and nutrition level have been given consistent with the animals’ habit and natural behavior. Animal keepers always maintain their self-cleanliness. GLZ has provided cages according to the technical instruction of constructing ideal cages, but the cages in TSTJ are worrying as they are not consistent with standard, rusty, and damaged, and animals have no partner. Some animals in GLZ are often sick, whereas some animals in TSTJ are dead due to poor maintenance. The iron pillars of cages restrict animal behavior in TSTJ so that they have not had freedom to behave normally yet, whereas, in GLZ, they can move freely in original habitat. The animals in the two zoos have not been free from disruption, stress, and pressure due to the passing over vehicles.

**Conclusion:**

There should be strategic communication, information, and education, community development, and law enforcement for the animal welfare.

## Introduction

Zoo has controversial label, as, on the one hand, it can attract tourists’ enthusiasm to visit, but a large number of visitors, on the other hand, can be a dangerous threat against the animals’ life sustainability when animal welfare is not prioritized [1,2]. Animal welfare is included into one of Sustainable Development Goals, targeted to be achieved up to 2030: Protecting, recovering, and promoting the utilization of ecosystem sustainability; managing forest, fighting against and stopping land degradation, and stopping biodiversity loss [3].

The framework for the analysis of animal welfare is the five freedoms principles including freedom from hunger and thirst; freedom from discomfort; freedom from pain, injury, and disease; freedom to behave normally; and freedom from fear and distress [4-8]. Animal welfare is an expression pertaining to morale intended to provoke human beings to treat animals wisely as the God’s creature and to develop the attitude of appreciating either domesticated or wild animal in nature [9-12]. Several countries have developed policy related to animal welfare: European countries [13,14] and United States of America [15], etc. Meanwhile, in Indonesia, it has been governed in the Republic of Indonesia’s Government Regulation Number 95 of 2012 about Veterinary Community and Animal Welfare.

The main functions of zoo are conservation, education, and research [6-18]. The zoo organized based on the five freedoms principles, and internal regulation gets good image because the animals can grow and develop without worry about extinction [19-22]. As legitimacy, the Zoo’s internal regulations including Standard Operating Procedure (SOP), manual and technical instruction about animal treatment and prohibition for visitors confirm that organizer, visitor, and stakeholders or zoo agents should comply with and apply them [23]. Unconscious motives, discursive consciousness, and practical consciousness implicitly affect the zoo agent’s action in bringing the animal welfare into reality [23-26].

However, zoo agents have not apparently understood and applied yet the animal welfare [27,28]. It can be seen from violence or negligence against animals occurring in many countries including Indonesia [29,30]. The massive news coverage by a variety of local and international mass media related to the death of animal collection in Surabaya zoo during 2013-2014 and the death of Sumatera elephant in Bandung zoo due to pulmonary disease and poor feed management have harmed the zoos’ reputation. Animal welfare issue is interesting to study such as zoonoses aspect [31-33], assessment [34,35], and problems in cattle [36-38], however, animal welfare is not only limited to biological aspect but also to social aspect [39-42]. This research aimed to compare the agents’ action in the two zoos: Taman Satwa Taru Jurug (thereafter called TSTJ) or Surakarta Zoo and Gembira Loka Zoo (GLZ) of Yogyakarta Indonesia, in applying the five freedoms principles for the animal welfare.

## Materials and Methods

### Ethical approval

To protect the zoo agents as the subject of research, all data and information obtained from the informant are safeguarded for their confidentiality and only used for research purpose [43].

### Study design and area

This research was conducted from July 2016 to September 2016 in TSTJ and GLZs, those with their flora and fauna collections attracting the tourist visit in Central Java and Daerah Istimewa Yogyakarta, Indonesia. Informants in this qualitative research with exploratory approach were selected using purposive sampling technique, consisting of the agents of TSTJ and GLZs. There were 19 informants: 8 from TSTJ coded T and 10 from GLZs coded G. The informants from TSTJ included Operational Manager and Animal Husbandry (T1), 1 veterinary (T2), 3 animal keepers for mammal, avis, and reptile (T3a, T3b, and T3c), 1 seller (T4), and 2 visitors (T5a and T5b). Meanwhile, the informants from GLZ were the Head of Animal Maintenance Division (G1); Head of Nutrition and Animal Health Division (G2), 2 veterinaries (G3a and G3b); 3 animal keepers for mammal, avis, and reptile (G4a, G4b, and G4c), and 3 visitors (G5a, G5b, and G5c). The author also interviewed the representatives of Natural Resource Conservation Center (thereafter called BKSDA) of Central Java (B).

### Data collection

The data were collected through observation, in-depth interview, focus group discussion (FGD) and documentation. All informants participated in FGD conducted on September 2016.

### Data validity and reliability

Method and source triangulations were used to validate the data [44].

### Statistical analysis

Data were analyzed in-depth using an interactive model of analysis. The author selected, concentrated, simplified, and classified the data obtained in the field, and then presented it in the form of narrative text, table, chart, or figure, to be understandable and to draw a conclusion and to verify by reflecting the data again in the field [45].

## Results

### The existence of TSTJ and GLZs

#### TSTJ as a conservation institution

TSTJ is one of natural tourist objects in Surakarta containing a variety of animal and vegetation species. Flora collections of TSTJ become a potency to be developed into a natural laboratory and flora conservation land. In addition, there are opened and closed stages for animal shows, and baby zoo, natural pond with relatively quiet water utilized as water tourist object, and Bird Park and aquarium building despite some improvement and collection increases needed. Considering the document of TSTJ Local Company’s management in 2015, the collection of animals consists of 442 animals: 275 protected and 167 unprotected by law and 15 taxidermic animals in 2016.

The types of zoo protected by law including mammal, avis, primate, and reptile demonstrated are, among others, 4 black bears (*Helarctos* sp.), 3 elephants (*Elephas maximus*), 1 antelope (*Mutiacus muntjak*), 10 land kangaroos (*Macropus*), 9 porcupines (*Hystrix* sp.), 4 Sumatran tigers (*Panthera tigris sumatrae*), 12 timorensis deer (*Cervus timorensis*), 1 lion (*Panthera leo*), 1 hippopotamus (*Hippopotamus amphibius*), 11 green peacocks (*Pavo muticus*), 3 pelicans (*Pelecanus* sp.), 4 crocodiles (*Crocodylus porosus*), and 2 sinyulong (*Tomistoma schlegelii*). Meanwhile, the animals unprotected by law are among others: 6 camels (*Camelus dromedarius*), 1 green peacock (*Pavo cristatus*), 14 Javanese monkeys (*Macaca fascicularis*), 4 python snakes (*Python reticulatus*), etc. Meanwhile, the collection of taxidermic animals consists of 15:1 elephants (*E. maximus*), 2 Sumatera tiger (*P. tigris sumatrae*), 1 leopards (*Panthera pardus*), etc.

#### GLZ as zoologicium museum

GLZ is located in Yogyakarta city, with 19.8 ha wide area, constituting a natural protection and conservation, research, education, and recreation medium that can attract many domestic and foreign tourists. There were 547,496 tourists in 2013, 1,796,865 in 2014, and 1,826,312 in 2015. In addition, to complete animal collection, many facilities and vehicles are offered including elephant attraction, riding elephant and camel elephant, rented bicycle, interaction with animal, catamaran boat, mobile transportation vehicles, boat, bumper boat, paddle boat, fish therapy, catching pond for children, ATV circuit, parking area, office buildings, toilet, mushola (small mosque), mayang tirta, restaurant, and education program held by GLZ in cooperation with Out of School Learning Department of Education Science Faculty of Yogyakarta State University. This activity targets Kindergarten, Elementary School, Junior High School, and Senior High School students.

As Zoologicium Museum, GLZ demonstrates protected animals, either alive or preserved (died). The collection of animals demonstrated includes mammal, pisces, avis, reptile, and amphibian, either native or foreign. The animals were obtained from submission, grant, and exchange, for example, Singapore zoo submitted some of its African penguin to GLZ demonstrated since May 2014, and GLZ sent two female Orangutans to Saigon zoo exchanged with Vietnam endemic animals. The zoning of animal cage distribution in GLZ is conducted by animal keeper considering location density, esthetics, and access to animal maintenance. Alive animals are put into the cage arranged in such a way that resembles natural habitat of those animals in wild nature, whereas the dead and preserved animals are demonstrated in GLZ museum, about 50 m from the entrance gate. The advantage of GLZ is that it has reptile and amphibian parks so that the visitors can see the reptiles and amphibians closely, freely, and securely.

Meanwhile, the animal collection demonstrated in GLZ as per January 2016 includes 329 mammals, 466 pisces, 387 avis, and 331 reptiles, and amphibians. Meanwhile, the dead and preserved animals are demonstrated in museum, including 2 Brazilian turtles (*Trachemys scripta elegan*), 2 proboscis monkeys (*Nasalis larvatus*), 1 sumatran owa (*Hylobates agilis*), 1 short-tailed macaque (*Macaca nemestrina*), and 2 sumatran tigers (*P. tigris sumatrae*), some types of insect: Dragonfly, beetle, scorpion, spider, cockroach, and uropygi, and some types of sea animals: Sand antlion, *Macrobrachium rosenbergii*, octopus, crab, sea worm, sea snake, jellyfish, mollusk, starfish, and sponge.

### The attempt of realizing animal welfare in TSTJ and GLZ

#### The application of freedom from hunger and thirst principle

Considering the result of observation, generally, the animals in TSTJ and GLZs seem to be healthy, given appropriate and sufficient food and drink. The management has fulfilled the animals’ feed and drink need viewed from feed preference, feed consumption, feed palatability, and feed preference index, and eating behavior. G1 stated that: In GLZ, we adjust animal feed preference with the animal’s original food in their habitat, for example, fruit and vegetables to keep the animal healthy (GLZ Yogyakarta, August 8, 2016).

Similarly, T1 said that: Standard feed and drink administration to animals is feed preference and variation for animals and the availability of clean feed and drinking water (TSTJ Surakarta, July 2, 2016).

G2 and G3a and G3b exemplify the agile wallaby, herbivore whose main feed is grass, but can consume grass root, leaves, and fruit. Green feed given includes kangkung (*Ipomea reptans)*, carrot (*Daucus carota)*, peanut leaf (*Arachis hypogaea)*, and cassava (*Manihot utilissima*). The nutrition content of green feed and the mean feed consumption per day for agile wallaby in wet and dry weights in GLZ is presented in Table-1.

**Table-1:** The nutrition content of green feed and the mean feed consumption per day for agile wallaby in wet and dry weights in GLZ.

Name of green feed	Water level	Carbohydrate	Protein	Fat	Fiber	Wet weight (kg/head/day)	Dry weight (kg/head/day)
Kangkung	90.20	5.00	3.00	0.30	1.00	0.23	0.02
Carrot	88.20	9.30	1.20	0.30	1.00	0.18	0.02
Peanut leaf	14.20	39.57	8.21	1.66	26.88	0.12	0.10
Cassava	65	30-36	1.5-1.2	0.2-0.4	1-3	0.06	0.02

Source: GLZ, September 2016. GLZ=Gembira Loka Zoo

**Figure-1:**
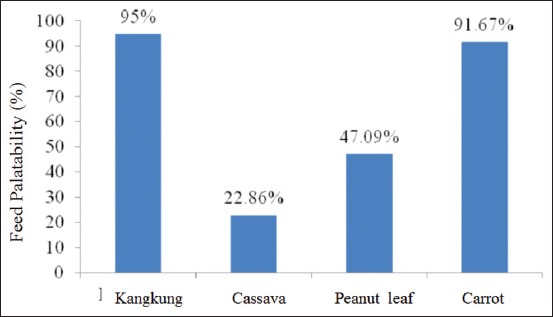
Percentage feed palatability of agile wallaby in Gembira Loka zoo.

The result of calculation shows that the percentage feed palatability of agile wallaby in GLZ is as follows: Kangkung of 95%, carrot of 91.67%, peanut leaf 47.09%, and cassava 22.86% is presented in Figure-1.

Considering the calculation using Neu method, according to G3a, it can be obtained the feed preference of wallaby successively as follows: Kangkung, carrot, peanut leaf, and cassava. Kangkung (*I. reptans)* has the highest palatability and feed preference index because wallaby likes green feed, with high water level and softer stem. Meanwhile, G3b stated that some food’s preference level will improve when its nutrition is adequate and animals like certain food from habit (GLZ Yogyakarta, August 8, 2016).

G4a argued that: Agile wallaby prefers feed containing much more water and mucus, like kangkung, because this content facilitates wallaby to digest the food (GLZ Yogyakarta, August 9, 2016).

Eating behavior of agile wallaby before, during, and after meal is presented in Table-2.

**Table-2:** Eating behavior of agile wallaby in GLZ.

Time	Hour	Behavior	Factor
Before meal	Before 10.30 am-01.00 pm	Sleeping, taking a rest, playing, walking around to look for food in the cage	
During meal	10.30 am-1.00 pm	Selecting the type of food by approaching and smelling the food container first, and then trying and tasting the food If it likes the food, it will take, chew, and ruminate it using its mouth, while its front legs hold and put the food into its mouth If it does not like the food, it will approach other food	The feed provided is still fresh It spent time by taking a rest when it is rain When the rain subsides, it continues its eating behavior
After meal	13.00-14.00 pm	Its eating behavior decreases to take a rest	
Taking a rest	14.00-15.30 pm	Eating activity increases, but then decreases When seeing many visitors, wallaby will approach them because some of them give food by throwing it into the cage	

Source: GLZ, September 2016. GLZ=Gembira Loka Zoo

**Figure-2:**
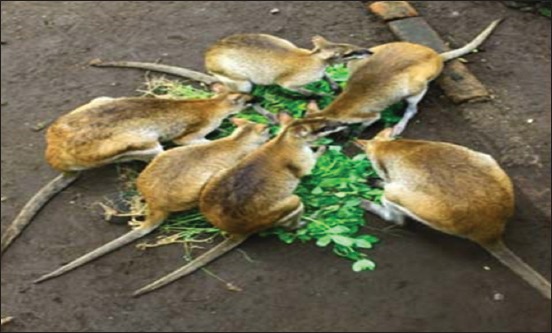
Eating behavior of agile wallaby in Gembira Loka zoo.

Wallaby’s eating behavior in group can be seen in Figure-2.

During FGD in TSTJ, T2 argued that: The time taken by the animal to eat is dependent on its species, physiological status (e.g., growth, end-period of pregnancy, lactation, and not pregnant, not lactating animals, and adult animal), and food type, and supply. Hence, the eating time of agile wallaby is dependent on the food supply provided by the management. Meanwhile, the effective time the wallaby takes to eat is when the food supply is sufficed. During its meal time, wallaby sometimes seems to vomit the food onto the floor, and then eat the food again (TSTJ Surakarta, September 2016).

The result of an interview with T3a and observation on TSTJ shows that in fact, male wallaby eats more than the female one. Female wallaby is carrying on its baby in its pouch, thereby decreasing its eating behavior and taking a rest more frequently than eating behavior. After eating, agile wallaby will leave its food container, take a rest, play, drink, and clean its hair by means of licking it. This activity occurs more frequently at the days, after having meal while taking shelter under a tree. T3a said that: During resting, when it sees many visitors, wallaby will walk approaching the visitors, because some visitors give it food such as peanut, banana, and leaves fell around the area out of the wallaby’s cage by throwing them into the cage. It changes its eating behavior (TSTJ Surakarta, July 4, 2016).

Animal keepers do the duty of maintaining agile wallaby in TSTJ and GLZ is by cleaning the cage, sweep it, and cleaning food and drink. The agile wallaby’s health is monitored once a week, but its condition is always monitored from outside cage daily.

It can be stated that T1 and G1 have understood that food and drink are basic needs for animals as living organism so that they should be fulfilled well. However, some visitors break the zoo’s rule by giving the animal the food. It of course will impact on the animal’s eating behavior change, in relation to animal’s food supply and choice, T1 and G2 recommended to prohibit the visitors from giving human food to the animal, such as processed food containing oil, salt, and sugar, and drink containing chemicals, as it is hazardous to the animal’s health. The visitors are recommended to buy the food provided by the management, such as vegetables, fruits, and fresh leaves.

#### The application of freedom from discomfort principle

The arrangement of animal’s cage or living environment in GLZ can be seen in Table-3.

**Table-3:** The arrangement of animal’s cage or living environment in GLZ.

Goals	Rationale	Infrastructure	Example
Animal comfort	Every animal’s need	Puddle pond Facilities of climbing to three directions (upward, lateral, and downward)	Animal usually puddling such as hippopotamus animal having climbing habits such as owa and monkey
The opportunity of moving according to the condition it experiences and its health is ensured	Adequate temperature, sunlight, air supply/ventilation Noise level in the cage is adjusted with the original habitat	Appropriate shelter The place with necessary heat	Animals accustomed to sunbath such as turtle, crocodile, and komodo
Healthy and natural environment	Water puddle can be the germ and disease nest	Sanitation and drainage	All water channels are maintained and cleaned periodically

Source: GLZ, September 2016. GLZ=Gembira Loka Zoo

G1 stated that: Particularly for the animals put into enclosure, the cage is designed in such a way using materials and equipment not harmful to the animals to mitigate the injury risk in animals. The cage also has a room designed to enable the animals to isolate themselves during quarrel between animals.

Meanwhile, G4a, G4b, and G4c argued that: We take care of the animals put into enclosure to prevent them from quarrel and injuring others, and monitor them according to animal keeper’s duty in each unit (GLZ Yogyakarta, September 3, 2016).

Considering the result of observation, it can be found that overall, agile wallaby can adapt to environment temperature because the agile wallaby’s cage in GLZ is constructed from wall with wire on its upper part enabling the visitors to see the agile wallaby. The cage is 4 m × 4 m wide containing 8 wallabies: 2 males, 4 females, and 2 baby wallabies. The floor is made of soil, but some of it is made of cement. There is shelter made of wood, cover, food, and drink container in it.

**Figure-3:**
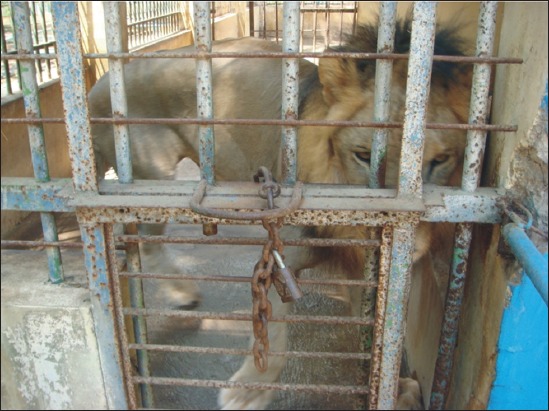
The lion encaged in the cage in Taman Satwa Taru Jurug.

On the contrary, in TSTJ, many animal cages are damaged even have not fulfilled yet the animals’ need for avoiding stress, thus requiring repairing. A number of animal cages are worrying, as they are not ideal, not standardized, rusty, and damaged. For example, lion’s cage that should be 4 m × 3 m wide is still below the standard in TSTJ. T3b did not want to answer related the condition of lion cage and recommended the author to ask it to T1 directly. Figure-3 shows a lion encaged in the cage in TSTJ.

The condition of animals in TSTJ is fairly worrying because a number of animals do not have friend or partner in the cage. Meanwhile, partner plays a very important part in animal reproduction. The absence of friend or partner makes the animal stressed as well. Regarding the absence of animal partner, T1 said that: Indonesian Zoo Association (Perhimpunan Kebun Binatang Se-Indonesia = PKBSI) has reported TSTJ to President Joko Widodo and it is followed up with the plan of revitalizing TSTJ. In Indonesia, there are 13 zoos managed by Local Government, but on average, their condition is worrying. For that reason, TSTJ becomes the pilot project of conservation institution revitalization. TSTJ has not been able to conduct revitalization so far due to limited budget (TSTJ Surakarta, July 9, 2016).

#### The application of freedom from pain, injury, and disease principle

Animal health in GLZ becomes the responsibility of Nutrition and Animal Health Division Head constituting a veterinary helped with other 4 veterinaries, 3 staffs for cleaning the quarantine cage, and 6 staffs in charge of preparing feed. The activity of maintaining the animal welfare in GLZ is presented in Table-4.

**Table-4:** The activity of maintaining animal health in GLZ.

Activity	Objective	Time
Animal vaccination	To improve the animal’s immunity to prevent vulnerability to certain disease	Once a year
Disinfection or spraying the animal cage with disinfectant	To prevent the transmission of disease to other animals, visitor, or animal keeper	Once a week
Treating the animal developing health disorder	To ensure the health of animal demonstrated	Incidental

Source: GLZ, September 2016. GLZ=Gembira Loka Zoo

G1 stated that: The attempt of maintaining animal health is conducted by monitoring or checking the animal condition. Animal condition, health, and behavior in GLZ are examined at least twice a day by the responsible animal keepers. The apparently sick animal is separated immediately and brought into the quarantine to get some treatment. The treatment of animal is adjusted with standard animal health (GLZ Yogyakarta, September 3, 2016).

Meanwhile, the animals in TSTJ had not enjoyed yet the freedom from pain, injury, and disease, despite veterinaries responsible for treating them routinely. The death of several animals in the past few years shows the worrying fact. On February 20, 2014, a 3-day-old baby camel died due to hypothermia, on May 28, 2014, female orangutan named Pebi died with dysentery diagnosis, on June 11, 2014, male orangutan named Kirno died with hepatitis diagnosis, followed with a 19-year-old African lion that died on June 26, 2014; this orangutan named Ony was sent from Surabaya zoo. The case of Sumatran tiger’s death has been submitted clinically and medically to Gadjah Mada University, and then BKSDA investigated the case from other aspects including board of directors, animal keepers, and veterinaries, related to the condition of animals, and feeding pattern. B stated that: The case of Sumatran tiger death is due to less varying food despite appropriate quantity of food (3.5-4 kg chicken). However, it cannot be found certainly whether or not the clean and hygienic cement floor affects the animal health and death. In addition, TSTJ organizer recognizes the adjustment of management due to limited budget (TSTJ Surakarta, July 9, 2016).

BKSDA’s findings had been reported to the Republic of Indonesia’s Living Environment and Forestry Ministry on January 5, 2015. BKSDA of Central Java had sent reprimand to TSTJ to prevent the animal death from recurring because, during 2014, some animals have died in TSTJ. Therefore, TSTJ should evaluate and developed zoo governance comprehensively.

#### The application of freedom to behave normally

**Figure-4:**
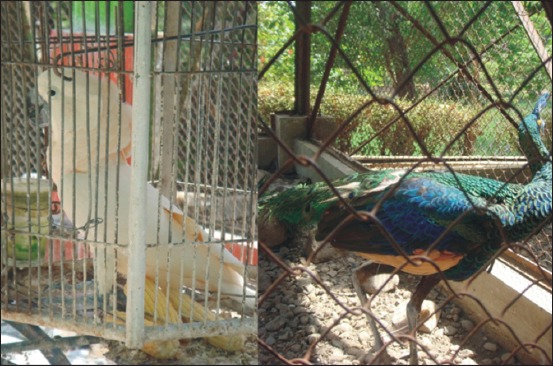
Cockatoo and peacock birds circumscribed in the cage in Taman Satwa Taru Jurug.

Animal collection in TSTJ is still difficult to get the freedom to behave normally like those in their habitat because TSTJ seems to be an animal contained circumscribed with iron pillars so that animals have no opportunity of interacting with other animal corresponding to their natural habitat. However, T3b did not want to answer when the author verified this issue. Figure-4 shows cockatoo and peacock birds circumscribed in the cage in TSTJ.

The condition of gardens existing in TSTJ supports the existence of animal, meanwhile, the visitors enjoying the animal diversity feel and get inadequate experience with interacting directly with the green natural tour circumstance in the zoo. It is because there is no circulation arrangement, no indoor and outdoor spatial layout, and local environment potency development. T5a stated that animal is a living organism entitled to move freely in its environment, whereas T5b answered that animal is God’s creature, of course, entitled to live comfortably and securely. G5a, G5b, and G5c stated that animal has feeling and needs affection. Hence, animal can feel violent treatment; therefore, we should love animal.

However, the collection of animal in GLZ can feel the freedom to behave normally or naturally just like in their nature and original habitat. It is indicated with the setting of cage making the animal live comfortably and undertaking their natural activities just like in wild nature. The author observed that pelican, for example, is given a wide, opened, and nonroof caged, surrounding with trees and the combination of land, water, or fish pond as the place to look for food, even stones and woods to take shelter are provided as well. G1 said that: The animal vulnerable to certain weather is adjusted with the weather, for example, *Arctictis binturong* is released lately at the day from accommodation cage in cloudy weather, and when it is rain at the day or evening, it will be led to accommodation cage (GLZ Yogyakarta, September 3, 2016).

GLZ is an area with distinctive microclimate in Yogyakarta City. The existence of vegetation has supported the created microclimate and sub-microclimate creating the habitat for a variety of wild animals, particularly the animals from insect, reptile, and avis. The members of avis class are wild animals often found in the location. Some vegetation grow in such the area so that forest ecosystem is created and the birds utilize the existing vegetation to look for food, take a rest, take shelter, and proliferate. The wild bird life in the area become distinctive attraction in GLZ, but the presence of wild bird and reptile in the area is threatened with hunting activity using air rifle, glue, and net. G4b and G4c suggest it when they brought the author to that area.

#### The application of freedom from fear and distress principle

The location of wallaby cage in GLZ is fairly close to the road crossed by the visitors, and there is a white tiger cage besides it so that many visitors stop by there. It disturbs the activity of agile wallaby. However, GLZ has prepared the animal for adapting to the visitors’ crowd so that they will not be stressed and afraid of the visitors. There is a procedure to prevent or to minimize the animal’s stress and fear, according to G1. It is presented in Table-5.

**Table-5:** The procedure to prevent and to minimize stress and fear in the animals in GLZ.

Procedure	Objective
The newly coming animal is put into the closed quarantine and cannot be seen by the visitors	To make the animal not afraid of and recognizing its new location and habitat in the zoo gradually
Interaction between animal and tamer, to make the animal accustomed with the existence of human being	To make the animal not afraid and stressed
Animal begins to be demonstrated but with some distance to the visitors, and the cage is equipped with hiding place	To give the animal the opportunity of hiding when it feels afraid of visitors

Source: GLZ, September 2016. GLZ=Gembira Loka Zoo

The collection of animal in TSTJ and GLZ can be stated as having no freedom from fear and distress because of the sound of cars and motors passing over around and outside the Zoo. T4 stated that during school vacation, many visitors come to TSTJ by their private vehicle, buy food, drink, and souvenir here. As we know, TSTJ and GLZs are located in the main ways of Surakarta and Yogyakarta, so that the animals do not have privacy and always be disturbed by vehicles, whistle, clapping, and noise of visitors.

## Discussions

A zoo governance system creates a structural principle showing the agent-structure relation of zoo [25]. As the conservation institution, TSTJ and GLZs mention and devise the animal welfare object, and animal as the subject of animal welfare. World Association of Zoos and Aquarium, South East Asian Zoo Association, Indonesian Zoo Association (PKBSI), and internal regulation of zoo dominate organizer, visitor, and stakeholders related to their knowledge, attitude, and action to realize animal welfare. Those institutions become the pioneer in introducing the term “the five freedoms” to the public as an international method and determining that whoever having an animal is responsible for ensuring that it is in welfare condition [9,12].

TSTJ and GLZs have held training about animal welfare including five freedoms principles, cage condition, food preference (food consumption, food palatability and food preference index, and eating behavior), isolation and quarantine of animal, and animal health care for organizers, and animal keepers. It means that they have had knowledge and information and discursive consciousness regarding this. However, in the structure of zoo, the action of ensuring the animal in welfare condition is affected by discursive consciousness referring to the zoo agents’ capacity of reflecting and clarifying the action done [46,47]. Zoo agent knows that wild animals have function for their life and habitat. Therefore, it is better for them to be released and to live in their habitat than to be circumscribed in the cage, to create natural balance, and beauty to maintain the worlds’ lung [48].

The aspect of animal freedom is also emphasized because animal needs freedom and natural proliferation [49]. Human being is responsible for the animal’s life. Government, community, and individual have responsibility and should ensure that animal may not be treated violently because animal is God’s creature to be used and conserved by human being. However, there is still a chain binding the monkey’s neck in zoo, and it is compelled to entertain the visitors, while it is an unpleasant thing [50].

The visitors of zoo are prohibited from disturbing and feeding the animal haphazardly, touching or disturbing animal, disposing rubbish, etc. [51,52]. However, not all visitors of TSTJ and GLZ obey the prohibition. The agent has determined the method and frequency of feeding, nutrition administration and nutrition level adjusted with the animal’s habit and natural behavior, season, and animal type. The food and drink portion provided has been adjusted with individual animal’s need [53]. Clean water for animal drink is always changed, and its container is maintained for cleanliness by washing it routinely to prevent the development of disease. The animal food is served in first-rate condition and its condition is maintained to prevent humidity or wetness because it will be vulnerable to fungus and insect or other pest contaminations [51,54]. Remnants of food are taken and cleaned to keep the cage clean, and the cage is checked in the morning and evening to find out whether or not the food addition is required. In addition, animal keepers in TSTJ and GLZs always maintain their self-cleanliness according to the specified standard cleanliness in feeding the animal. It is intended to avoid the cross-contamination from the tools and food containers used [55].

Cage is the facility provided in the zoo, but the condition of animal cages in TSTJ is worrying, as they are not ideal, not standardized, rusty, and damaged. Many animals have no partner in TSTJ so that they are stressed and no reproduction occurs [47,56,57]. The collection of animals in GLZ seems to be more comfortable compared with that in TSTJ because the organizer of GLZ has provided cages according to SOP, manual and technical instruction about constructing the ideal cages. To bring the animal welfare into reality, the organizer of zoo should provide living environment consistent with the animal need to behave naturally. Natural habitat of animal should be prioritized, for example, shelter is provided according to the animal habitat [58].

The principle of freedom from pain, injury, and disease has been applied by the organizer but it has not been optimal yet so that some animals in GLZ are sick, even some in TSTJ dead in the past few years. The attempt of maintaining and taking care of the animal health should be consistent with the standard animal health, by means of monitoring or checking the condition of animal; the animal keeper check the animal health at least twice a day, and the animals apparently sick should be separated and brought into the quarantine to get special treatment [59].

Many animals in TSTJ have not gotten the freedom to behave normally because of the iron pillars of cage circumscribing their behavior, so that they cannot interact with other animals corresponding to their habitat. Meanwhile, the collection of animal in GLZ can feel more freedom to behave normally just like in their original habitat in the presence of cage making them living comfortably and doing such activities as looking for food, drink, and running around the wild nature [58]. The animals in the zoo should move freely in the wide environment enabling them to do natural movement to interact with other similar animals [8,10,37].

The animals in either TSTJ or GLZ have not been free from fear and distress so far, particularly because of so many vehicles passing over in the zoo area, so that they have no privacy. The animals are also disturbed with whistle, clapping, and noise of visitors. The visitors of zoo affect the animals, for example, they can make the animal distressed, neutral, and enriching, whereas the freedom from distressed and fear is the principle that should be fulfilled. Therefore, the zoo agent should ensure the animal condition and treatment corresponding to the rule to avoid the animal from boring, stress, and fear threats [60-63].

Considering the discussion above, it can be stated that the agent applies knowledge, attitude, and discursive consciousness less optimally in relation to the five freedoms principle with the real action to make the animal in well-being condition because the agent only wrestles with the prohibition of feeding and giving drink and the maintenance of cage cleanliness only.

## Conclusions

Zoo agent should improve communication, information, and education, and enforce the five freedoms principle sustainably to bring the animal welfare into reality. The animal’s right to freedom from distress and to live prosperously should be respected. In addition, community development can improve the appreciation to animal and natural environment, and law enforcement can be conducted to implement the policy effectively.

## Authors’ Contributions

AD, RBS, and SZ had made substantial contributions to conception, design, data collection, analysis, and interpretation of data; drafting the article, revising it critically for important intellectual content; and final approval of the version to be published.

## References

[1] Beck B.B, Norton B.G, Hutchins M, Maple T.L (1995). Reintroduction, zoos, conservation and animal welfare. Ethics on the Ark: Zoos, Animal Welfare and Wildlife Conservation.

[2] Patrick P.G, Matthews C.E, Ayers D.F, Tunnicliffe S.D (2007). Conservation and education: Prominent themes in zoo mission statement. J. Environ. Educ.

[3] United Nations (2016). The Sustainable Development Goals Report 2016.

[4] D'Eath R.B, Tolkamp B.J, Kynazakis L, Lawrence A.B (2009). Freedom from hunger and prevent obesity: The animal welfare implications of reducing food quantity or quality. Anim. Behav.

[5] McCulloch S.P (2013). A critique of FAWC's five freedoms as a framework for the analysis of animal welfare. J. Agric. Environ. Ethics.

[6] de Vries M, Bokkers E.A.M, van Schaik G, Botreau R, Engel B, Dijkstra T, de Boer J.M (2013). Evaluating the results of the welfare quality multi-criteria evaluation model for classification of dairy cattle welfare at the herd level. Int. J. Dairy Sci.

[7] Anonymous (2014). The Five Freedoms;Royal Society for the Prevention of Cruelty to Animals: Australia.

[8] Mellor D.J (2016). Updating animal welfare thinking: Moving beyond the “five freedoms” towards “a life worth living”. Animals.

[9] Haynes R.P (2008). Animal Welfare: Competing Conceptions and their Ethical Implications.

[10] Beaver B.V, Breed M.D, Moore J (2010). Welfare of animals: Introduction. Encyclopedia of Animal Behavior.

[11] Kagan R, Vease J, Kleiman D.G, Thompson K.V, Baer C.K (2010). Challenges of zoo animal welfare. Wild Mammals in Captivity Principles and Techniques for Zoo Management.

[12] Appleby M.C, Mench J.A, Olsson J.A.S, Hughes B.A (2011). Animal Welfare.

[13] Ingenbleek P.T.M, Immink V.M, Spoolder H.A.M, Bokma M.H, Keeling L.J (2012). EU animal welfare policy: Developing a comprehensive policy framework. Food Polish.

[14] Jacques S (2014). Science and animal welfare in France and European union: Rules, constraints, achievements. Meat Sci.

[15] Bradfield J.F, Bennett B.T, Gillett C.S (2014). Oversight of research animal welfare in the United States. Laboratory Animals Regulations and Recommendations for Global Collaborative Research.

[16] Tribe A (2001). Wildlife Tourism Research Report Series: No. 14. Status Assessment of Wildlife Tourism in Australia Series.

[17] Catibog-Sinha C (2008). Zoo tourism: Biodiversity conservation through tourism. J. Ecotourism.

[18] Patrick P.G, Tunnicliffe S.D (2013). Zoo Talk.

[19] Swaisgood R.R, Shepherdson D.J (2005). Scientific approaches to enrichment and stereotypies in zoo animals: What's been done and where should we go next?. Zoo. Biol.

[20] WAZA (World Association of Zoos and Aquariums (2005). Building a Future for Wildlife: The World Zoo and Aquarium Conservation Strategy.

[21] Rabb G.B, Saunders C.D (2005). The future of zoos and aquariums: Conservation and caring. Int. Zoo. Yearb.

[22] Hosey G, Melfi V, Pankhurst S (2013). Zoo Animals: Behavior, Management and Welfare.

[23] Stevens P.M.C, McAlister E (2003). Ethics in zoos. Int. Zoo Yearb.

[24] Giddens A (1984). The Constitution of Society: Outline of The Theory of Structuration.

[25] Stones R (2005). Structuration Theory.

[26] Craib I (2011). Anthony Giddens (Routledge Revivals).

[27] Bell C.E (2001). Encyclopedia of the World's Zoos.

[28] Club R, Mason G (2002). A Review of the Welfare of Zoo Elephants in Europe.

[29] Hewson C.J (2003). Can we assess welfare?. Can. Vet. J.

[30] Grandin T (2014). Animal welfare and society concerns finding the missing link. Meat Sci.

[31] Kumar H.B.C, Lokesha K.M, Madhavaprasad C.B, Shilpa V.T, Karabasanavar N.S, Kumar A (2013). Occupational zoonoses in zoo and wildlife veterinarians in India. Vet. World.

[32] Emmanuel J, Awosanya H.O, Akande H.O (2015). Animal health care seeking behavior of pets or livestock owners and knowledge and awareness on zoonoses in a university community. Vet. World.

[33] World Health Organization (2015). Zoonoses.

[34] Velarde A, Dalmau A (2012). Animal welfare assessment at slaughter in Europe: Moving from inputs to outputs. Meat Sci.

[35] Andreasen S.N, Sandoe P, Forkman B (2014). Can animal-based welfare assessment be simplified?A comparison of welfare quality protocol for dairy cattle and the simpler and less time consuming protocol developed by the Danish Cattle Federation. Anim. Welf.

[36] Hemsworth P.H, Rice M, Karlen M.G, Calleja L, Barnett J.L, Nash J, Coleman G.J (2011). Human animal interactions at abattoirs: Relationships between handling and animal stress in sheep and cattle. Appl. Anim. Behav. Sci.

[37] Munoz D, Strappini A, Gallo C (2012). Animal welfare indicators to detect problems in cattle stunning box. Arch. Med. Vet.

[38] Schwartzkopf-Genswein K.S, Faucitano L, Dodger S, Shand P, Gonzales L.A, Crowe T.G (2012). Road transport of cattle, swine and poultry in North America, and its impact on animal welfare and meat quality: A review. Meat. Sci.

[39] Webster J (2005). Animal Welfare Limping Towards Eden: A Practical Approach to Redressing the Problem of Our Dominion over the Animals.

[40] Fraser D (2008). Understanding Animal Welfare: The Science in Its Cultural Context.

[41] Ohl F, van der Staay F.J (2012). Animal welfare: At the interface between science and society. Vet. J.

[42] Špinka M (2012). Social dimension of emotions and its implication for animal welfare. Anim. Behav. Sci.

[43] Patton M.Q (2015). Integrating theory and practice. Qualitative Research and Evaluation Methods.

[44] Creswell J.W (2008). Educational research planning, conducting and evaluating quantitative and qualitative research.

[45] Miles M.B, Huberman A.M, Saldana J (2014). Qualitative Data Analysis: A Methods Sourcebook.

[46] Koknaroglu H, Akunal T (2013). Animal welfare: An animal science approach. Meat Sci.

[47] Grandin T (2010b). Improving animal welfare: A practical approach.

[48] Kagan R, Carter S, Allard S (2015). A universal animal welfare framework for zoos. J. Appl. Anim. Welfare Sci.

[49] Spencer T.E (2013). Early pregnancy: Concepts, challenges and potential solutions. Anim. Front.

[50] Hanson E (2002). Animal Attractions: Nature on Display in American Zoos.

[51] Broom D.M (2010). Animal welfare: An aspect of care, sustainability, and food quality required by the public. J. Vet. Med. Educ.

[52] Wells D.L (2005). A note on the influence of visitors on the behavior and welfare of zoo-housed gorillas. Appl. Anim. Behav. Sci.

[53] Grandin T (2012). Developing measures to audit welfare of cattle and pigs at slaughter. Anim. Welf.

[54] Grandin T (2005). Maintenance of good animal welfare standards in beef plants by use of auditing programs. J. Am. Vet. Med. Assoc.

[55] Swai E.S, Schoonman L, Daborn C.J (2010). Knowledge and attitude towards zoonose among animal health workers and livestock keepers in Arusha and Tanga, Tanzania. Tanzan. J. Health Res.

[56] Gregory N.G (2004). Physiology and Behavior of Animal Suffering.

[57] Koene P (2013). Behavioral ecology of captive species: Using behavioral adaptations to assess and enhance welfare of nonhuman zoo animals. J. Appl. Anim. Welf. Sci.

[58] Mellor D.J (2015). Positive welfare states and promoting environment-focused and animal-to-animal interactive behaviors. N. Z. Vet. J.

[59] Jhon K, Kazwala R, Mfinanga G.S (2007). Knowledge of causes, clinical features and diagnosis of common zoo-noses among medical practitioners in Tanzania. BMC. Infect. Dis.

[60] Grandin T (1997). Assessment of stress during handling and transport. J. Anim. Sci.

[61] Mader T.L, Davis M.S, Brown-Brandl T (2005). Environmental factors influencing heat stress in feedlot cattle. J. Anim. Sci.

[62] Moss A, Esson M (2010). Visitor interest in zoo animals and the implications for collection planning and zoo education programmes. Zoo Biol.

[63] Choo Y, Todd P.A, Li D (2011). Visitor effects on zoo orangutan in two novel, naturalistic enclosure. Anim. Behav. Sci.

